# Design and characterization of AgVO_3_-HAP/GO@PCL ceramic-based scaffolds for enhanced wound healing and tissue regeneration

**DOI:** 10.1007/s10856-025-06907-1

**Published:** 2025-06-25

**Authors:** Hagar M. Mahdy, Hanan Hendawy, Yehia M. Abbas, El-shazly M. Duraia

**Affiliations:** 1 Physics Department, Faculty of Science, Suez Canal University, Ismailia, 41522 Egypt; 2 Department of Veterinary Surgery, Faculty of Veterinary Medicine, Suez Canal University, Ismailia, 41522 Egypt

## Abstract

Rapid, infection-free wound healing remains a critical challenge in regenerative medicine. This study presents the fabrication and evaluation of multifunctional electrospun polycaprolactone (PCL)-based scaffolds incorporating silver vanadate (AgVO_3_), hydroxyapatite (HAp), and graphene oxide (GO) for advanced wound care applications. AgVO_3_ offers potent antibacterial properties, HAp supports osteogenic and regenerative activities and GO enhances both mechanical performance and cellular interactions. The scaffolds exhibited a highly porous nanofibrous structure, mimicking the extracellular matrix (ECM) and promoting cell attachment, migration, and nutrient exchange. Comprehensive physicochemical characterization using X-ray diffraction (XRD), Fourier-transform infrared spectroscopy (FTIR), Raman spectroscopy, and field-emission scanning electron microscopy (FE-SEM) confirmed the successful integration of the composite. Mechanical testing revealed that GO-containing scaffolds significantly improved stiffness, with AgVO_3_/GO@PCL and HAp/GO@PCL achieving Young’s moduli of 5.82 MPa and 4.36 MPa, respectively, which are substantially higher than that of neat PCL (1.39 MPa). In terms of flexibility, HAp/GO@PCL displayed the highest elongation at break (107.54%), indicating exceptional stretchability. The ultimate tensile strength was also enhanced in HAp@PCL (0.80 kJ/m^3^) and AgVO_3_/@PCL (0.88 kJ/m^3^), highlighting their capacity to resist mechanical stress during application. Contact angle measurements showed that the AgVO_3_-HAp/GO@PCL scaffold had the highest hydrophilicity (65.58° ± 5.97), compared to pure PCL (89.89° ± 3.70), indicating improved wettability, which is critical for fluid management and cell-material interactions at the wound interface. In vivo wound healing studies using a full-thickness rat model demonstrated that AgVO₃/GO@PCL scaffolds achieved 50% wound closure within 3 days, while AgVO₃-HAp/GO@PCL scaffolds facilitated complete re-epithelialization by day 14. Histological analysis confirmed enhanced collagen deposition and organized tissue architecture. The scaffolds also exhibited strong antibacterial activity, with large inhibition zones against *S. aureus* and *E. coli*. These findings position AgVO₃-HAp/GO@PCL scaffolds as promising candidates for next-generation wound dressings, offering a robust combination of mechanical resilience, bioactivity, antimicrobial efficacy, and moisture balance tailored for clinical wound-healing applications.

**Graphical Abstract:**

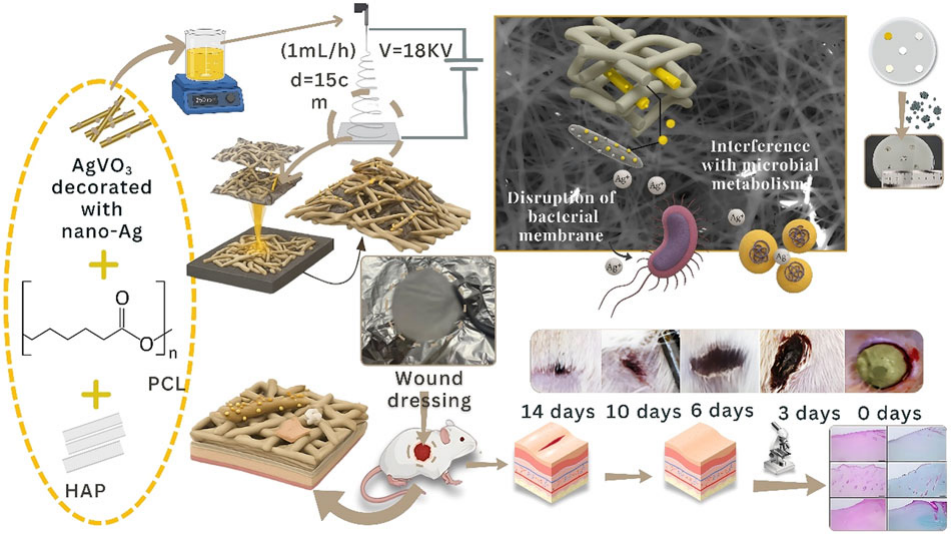

## Introduction

Wound healing is a dynamic biological process that can be disrupted by infection, chronic inflammation, or underlying conditions such as diabetes, obesity, and aging [1]. These factors contribute to delayed healing, an increased risk of complications, and prolonged patient recovery. Effective wound care requires materials that not only protect the wound but also actively support tissue regeneration and prevent infection [2].

Conventional wound dressings provide essential physical coverage but are often insufficient to address the multifactorial challenges associated with chronic, non-healing, or infected wounds. In recent years, advanced wound dressings incorporating bioactive and antimicrobial components have demonstrated improved therapeutic outcomes by actively promoting tissue regeneration and infection control [3]. However, the clinical implementation of these advanced materials remains limited because of high production costs, restricted availability, and disparities in healthcare access. These limitations are particularly pronounced in resource-constrained settings, where financial constraints and inadequate insurance coverage further restrict access to effective wound care solutions. Consequently, there is a critical need for the development of multifunctional, cost-effective wound dressings that are both clinically efficacious and widely accessible [4]. Ensuring the equitable distribution and affordability of advanced wound care technologies is essential for improving patient outcomes on a broader scale [5].

Electrospun scaffolds and hydrogels represent two major classes of wound-healing materials, each with distinct benefits. Hydrogels are known for their high-water content and ability to maintain a moist wound environment, which facilitates autolytic debridement and accelerates healing in superficial or exuding wounds [6]. However, they often lack mechanical strength and structural stability, limiting their application to larger or deeper wounds. In contrast, electrospun scaffolds, such as those fabricated in our study, provide a fibrous architecture that mimics the ECM, offering excellent mechanical integrity and a high surface area for cell attachment, proliferation, and tunable properties [7–9].

PCL, a biodegradable polyester, has garnered considerable attention in biomedical applications, particularly in wound healing. This polymer exhibits several advantageous properties that render it suitable for such applications. PCL demonstrates biocompatibility [10, 11] as it does not elicit adverse immune responses or exhibit toxicity upon contact with biological tissues [12]. The polymer undergoes gradual degradation within the body via hydrolysis of its ester linkages [13], providing temporary structural support to the wound site while being progressively replaced by native tissue throughout the healing process. The mechanical properties of PCL, including its strength and flexibility, make it an ideal candidate for fabricating various wound dressings, scaffolds, and implants [14]. Additionally, PCL functions as an effective carrier for the controlled release of bioactive molecules, such as growth factors, antimicrobial agents, and anti-inflammatory drugs, at the wound site. Incorporating these molecules into PCL-based matrices enables the modulation of their release kinetics, promoting wound healing and preventing infection. PCL also facilitates cellular processes crucial for tissue regeneration, including cell attachment, proliferation, and migration. The surface properties of PCL can be modified to enhance cell adhesion and promote interactions with key cell types involved in the healing process, such as fibroblasts and keratinocytes. Furthermore, PCL-based wound dressings provide a barrier against microbial contamination while maintaining gas exchange and moisture regulation, thereby creating an environment conducive to wound healing and protection from external pathogens.

Bioactive nanomaterials can be incorporated to enhance the therapeutic potential of PCL-based scaffolds. AgVO_3_, a vanadium-based nanomaterial, offers potent and sustained antimicrobial activity through the controlled release of silver ions, which disrupt bacterial membranes and interfere with microbial metabolism [15]. Beyond its antibacterial efficacy, AgVO_3_ attenuates proinflammatory cytokine production and promotes the proliferation of key skin cells, such as fibroblasts and keratinocytes [16], thereby contributing to a more balanced and regenerative wound microenvironment. HAp, a bioactive calcium phosphate ceramic, has long been used in bone repair; however, emerging evidence supports its role in soft tissue regeneration [17–19]. In cutaneous wounds, HAp enhances granulation tissue formation, stimulates angiogenesis, and supports ECM remodeling, all of which accelerate tissue repair. GO, a functionalized carbon nanomaterial, reinforces polymeric scaffolds by improving their tensile strength, elasticity, and surface area, while its oxygen-rich functional groups promote hydrophilicity and facilitate cellular adhesion, migration, and proliferation [20]. The integration of AgVO_3_, HAp, and GO into biodegradable PCL matrices enables the development of electrospun nanofibrous scaffolds that closely mimic the architecture of the ECM and address the multifactorial requirements of chronic and infected wounds. Such hybrid systems offer a compelling strategy for next-generation wound care by combining antimicrobial defense, bioactive regeneration, and structural performance within a single biomedical platform.

This study focuses on the fabrication and characterization of electrospun PCL-based scaffolds incorporating HAp, AgVO₃, and GO for multifunctional wound-dressing applications. The resulting scaffolds were evaluated for their structural, mechanical, antimicrobial, and wound-healing properties. This approach aims to create an advanced wound dressing capable of promoting tissue regeneration, managing infection, and providing mechanical support, which are key features for effective wound healing in complex clinical settings.

Electrospinning is a versatile and widely used fabrication technique in tissue engineering, known for producing nanofibrous scaffolds with a high surface area, tunable porosity, and mechanical resilience [21]. By applying a high-voltage electric field, ultrafine polymer fibers are generated and solidified through solvent evaporation, forming scaffold structures that mimic the native ECM [22]. These properties make electrospun scaffolds highly suitable for biomedical applications, such as wound healing, drug delivery, and tissue regeneration [23]. This comprehensive investigation aims to establish AgVO₃-HAP/GO@PCL scaffolds as promising ceramic-polymer hybrid materials for advanced wound care and regenerative medicine, offering enhanced structural integrity, infection control, and cellular functionality.

## Experimental work

### Preparation of scaffold

**Fig. 1:**
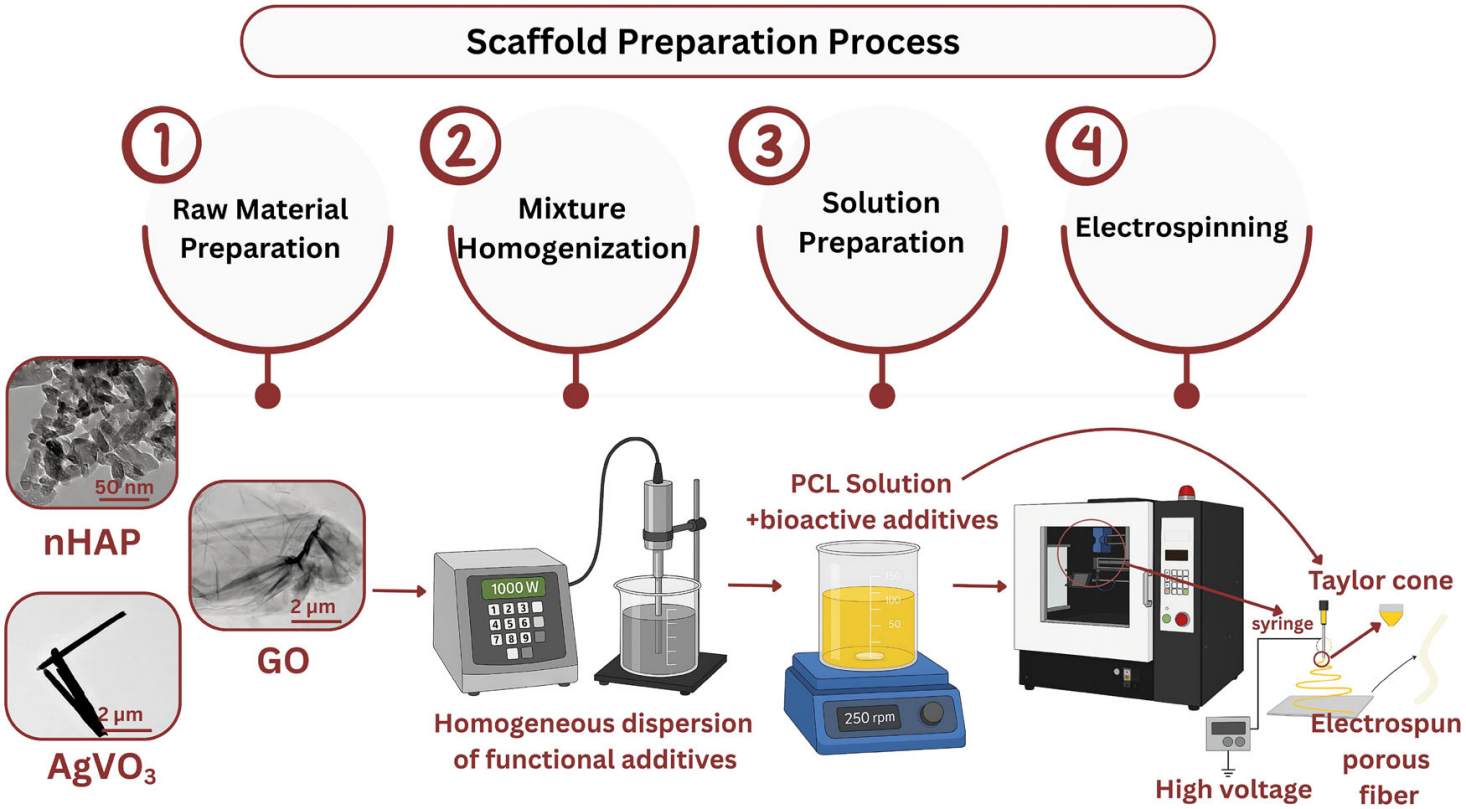
Stages of Scaffold preparation

The preparation of the seven samples involved four distinct stages, as shown in Fig. 1. The process began with the preparation of raw powders of GO, AgVO_3_, and HAp, followed by mixture preparation and scaffold fabrication. The fabricated scaffolds, including HAP@PCL, AgVO_3_@PCL, AgVO_3_-HAP@PCL, AgVO_3_/GO@PCL, HAP/GO@PCL, and AgVO_3_-HAP/GO@PCL, were systematically analyzed to assess their structural, mechanical, and biological properties.

#### Graphene oxide synthesis procedure

The detailed procedure for the synthesis of graphene oxide nanosheets can be found in our previous work [24]. In summary, GO nanosheets were synthesized using a modified Hummers’ method. Graphite flakes were mixed with sulfuric acid and sodium nitrate at low temperatures, followed by the gradual addition of potassium permanganate to maintain oxidation conditions. The reaction was further processed by adding water to induce an exothermic response, followed by hydrogen-peroxide treatment to achieve complete oxidation. The resulting suspension was filtered, washed with hydrochloric acid and deionized water until a neutral pH was obtained, and then dried under ambient conditions.

#### Synthesis of silver vanadate

Silver vanadate was synthesized by slowly adding a silver nitrate solution dropwise to an ammonium vanadate solution in a 1:1 molar ratio under continuous stirring at room temperature, while maintaining the pH between 4.6 and 5.8. The reaction resulted in a yellow precipitate, indicating the successful formation of the product. The mixture underwent hydrothermal treatment at 150 °C for 4 h, converting the precipitate into AgVO_3_. The product was thoroughly washed with ethanol and deionized water to remove impurities and dried at 60 °C overnight to preserve its structural integrity. The resulting material was then prepared for subsequent characterization and applications [25].

#### Hydroxyapatite preparation

Hydroxyapatite was synthesized by mixing 0.5 M calcium chloride (CaCl_2_) and 0.3 M sodium hydrogen phosphate (Na_2_HPO_4_) solutions under continuous stirring. Ammonia solution was gradually added to adjust the pH to 11. The resulting mixture was stirred continuously and allowed to age for 24 h. The precipitate was then filtered, thoroughly washed with distilled water to remove impurities, and dried at 55 ± 5 °C [26].

#### Preparation of composite mixtures

A uniform mixture was prepared at a weight ratio of 1:0.05 (AgVO_3_ to GO or HAp to GO) using a 1500 W ultrasonic probe (BEM-1500D). GO, which is known for its excellent water dispersion, was used without further modification. Specific additives were incorporated during the exfoliation process to investigate its effects. The sample, prepared as a 500 mL solution, was sonicated for 45 min using a probe equipped with a 2.5 cm diameter horn. This ensured the thorough mixing and optimal dispersion of the components.

#### Scaffold preparation

The PCL solution was prepared by dissolving 10 g of PCL pellets in 100 mL of a solvent mixture consisting of 66.7 mL chloroform and 33.3 mL methanol. Prepared samples were then incorporated into the PCL solution and stirred continuously overnight to ensure homogeneity. The resulting composite mixtures were loaded into a syringe pump for the electrospinning process. The operating parameters were set to a high voltage of 18 ± 0.1 kV, an injection rate of 1 mL/h, a distance of 15 cm between the electrode and the target, and a syringe needle size of 22φ [27].

### Characterization techniques

#### SXRD measurements

The crystallographic structures of the synthesized samples were examined using XRD on the MS beamline at the SESAME. Measurements were conducted within an angular range of 5° to 90°, using a step size of 0.05°, step time of 1 s, and wavelength of 0.81040 Å [28]. The MS beamline was equipped with a wiggler source, double-crystal Si (111) Kohzu monochromator with a sagittally bendable second crystal, and two rhodium-coated mirrors for beam conditioning. The beam flux was monitored in real time using an ionization chamber at the experimental station, allowing for scale factor corrections during extended measurement periods. The setup featured a two-circle vertical diffractometer with high-resolution encoders and a Pilatus 300 K area detector (172 µm pixel size) positioned 740.4 mm from the sample. At this distance, the detector covered a 2θ range of 6.4°. Diffraction patterns were collected in the 2θ range of 1.0° to 60.0°, with each frame captured over a 4-min exposure time.

The samples were loaded into polyimide capillaries mounted on a standard goniometer head with a capillary spinner for uniform rotation. For in situ XRD measurements during heating, an Oxford FMB gas blower was aligned beneath the capillaries, and the heat-time profiles were adjusted according to the experimental requirements. All XRD measurements were performed in the transmission mode (Debye-Scherrer geometry) at room temperature. Calibration was conducted using an NIST (640 f) silicon standard, and the wavelength was refined based on its lattice parameter. Diffraction images were captured every 6° and processed using an in-house Python script built on the pyFAI library for fast azimuthal integration to produce the final diffraction patterns.

The crystallinity index was calculated by separating the crystalline and amorphous contributions using peak fitting techniques.

1 $${\rm{CI}}=\frac{{A}_{c}}{{A}_{c}+{A}_{a}}\times 100$$

Where *A*_*C*_ is the area under the crystalline peaks and *A*_*a*_ is the area under the amorphous peaks.

The crystallite size and microstrain were calculated using the Williamson-Hall equation, as expressed in Eq. (2) [29].

2 $$\beta \cos \theta =\frac{k\lambda }{D}+4\varepsilon \sin \theta$$

where *β* is the integral breadth of the diffraction peak (radians), λ is the X-ray wavelength, and *k* is the shape factor of the crystallite.

#### FTIR and Raman measurements

Fourier-transform infrared spectroscopy (FTIR) was performed using a PerkinElmer 2000 spectrometer within the 4000–500 cm^−1^ range, with samples prepared on KBr substrates for enhanced signal detection. Raman spectra were obtained using an i-Raman Plus system with a 532 nm laser as the excitation source, enabling detailed analysis of vibrational modes and structural properties [30]. Using the calculated ID/IG ratio, in conjunction with the laser source energy (E_L_ = 2.33 eV), wavelength (λ_L_ = 532 nm), and laser power of 3 mW, it is possible to determine the crystallite size (La), average defect distance (L_D_), density of defects (n_D_), and in-plane sp² domain length (L_sp_²) using the following methods:

3 $${{\rm{L}}}_{{\rm{a}}}=\frac{(2.4\times {10}^{-10}\times {\lambda }_{l}^{4})}{(\frac{{I}_{D}}{{I}_{G}})}$$

4 $${{\rm{L}}}_{{\rm{D}}}=\surd \left(\frac{4.3\times {10}^{3}}{{E}_{L}^{4}\times \left(\frac{{I}_{D}}{{I}_{G}}\right)}\right)$$

5 $${{\rm{n}}}_{{\rm{D}}}=7.3\times {10}^{9}\times {E}_{L}^{4}\times \left(\frac{{I}_{D}}{{I}_{G}}\right)$$

6 $${{\rm{L}}}_{{\rm{sp}}2}=\frac{560}{{E}_{L}^{4}\times (\frac{{I}_{D}}{{I}_{G}})}$$

#### Morphology and roughness measurements

The surface morphology and roughness characteristics of the samples were examined using field-emission scanning electron microscopy (FE-SEM) with a QUANTA-FEG250 microscope (Netherlands). The acquired images were analyzed using the ImageJ software to extract detailed surface features and roughness parameters.

#### Mechanical testing

Rectangular specimens measuring 100 × 20 × 0.1 mm were prepared. Subsequently, stress/strain analysis was conducted by subjecting the nanofibers to a tensile force at a rate of 5 mm/min until failure, in accordance with the ASTM D882 standard protocol. To determine the standard deviation, measurements were replicated three times for each sample group.

#### Contact angle measurements

A contact angle analyzer (OCA15EC, DataPhysics, Germany) was employed to determine the contact angles of the electrospun scaffolds at ambient temperature. To ensure statistical reliability, each sample group underwent a minimum of three measurements to calculate the standard deviations.

#### Antibacterial activity test

The antibacterial activity of the samples was assessed against both gram-positive (Staphylococcus aureus (ATCC 25923)) and gram-negative (Escherichia coli (ATCC 25922)) bacteria under the same conditions. The standard agar plate assay was employed, and antibacterial effectiveness was evaluated using the “disc diffusion method” [31]. The inhibition zones were measured in triplicate after three days of incubation at 37 °C.

#### In vivo wound healing test (Surgical induction of skin wounds and innovative dressing management strategies)

This study utilized 24 male Sprague-Dawley (SD)rats. D. rats, aged 8 weeks and weighing between 250 and 300 g, were maintained under pathogen-free conditions and divided into eight groups with controls, each group containing three rats. The rats were housed in a controlled environment with temperatures set between 20 and 25 °C, relative humidity of 40–60%, and a 12-h light/dark cycle. The animals were provided ad libitum access to standard food and water. The animals were randomly assigned to eight groups of three animals each (*n* = 3).

Anesthesia was administered via intraperitoneal injection of ketamine and xylazine at a dosage of 0.2 mL per 100 g body weight. After depilation and disinfection with 70% ethanol, full-thickness wounds measuring 10 mm in diameter were created on the dorsal surface of the rats using a sterile skin biopsy punch to expose the dorsal muscle fascia. Treatment Protocols for the experimental groups

Daily measurements of the wound area and edge diameter were performed using a digital caliper both pre- and post-treatment. Wound edge contraction was calculated as the percentage reduction in the original wound diameter. Damaged areas were cleaned and debrided regularly before being covered with nanofibrous scaffolds. A nonadherent, nonocclusive dressing (Tri M Strip®) was applied to each wound, with dressing changes conducted carefully to maintain the integrity of healthy granulation tissue.

Following treatment, the rats were housed individually at an ambient temperature and monitored daily for two weeks. Wound healing was assessed at 3, 6, 10, and 14 days based on clinical parameters, including wound appearance, color, contraction, granulation tissue uniformity, epithelialization, and exudation, in addition to continuous recording of wound size. Observations were documented for statistical analysis, and digital photographs were captured to complement the data obtained. Before the detailed examination, the wounds were gently cleaned with sterile saline, and a tape measure was used as a calibration reference for photographs.

Animal experiments were conducted at Suez Canal University, Egypt, in accordance with ethical guidelines. Ethical approval for the study was granted by the Faculty of Veterinary Medicine’s Ethical Committee, Suez Canal University, Egypt (ethical approval ID: SCU-VET2024017).

#### Histological analysis

After the 14-day study period, the rats were euthanized, and skin tissue samples were preserved in 10% formalin for 24 h. The samples were then dehydrated with graded ethanol, embedded in paraffin, and sectioned into 5 μm slices using a microtome. The sections were deparaffinized and stained with hematoxylin and eosin (H&E) for general histology and Masson’s trichrome (MTC) for collagen evaluation and then analyzed under an optical microscope. Quantitative analysis was performed using the ImageJ software (NIH, USA). The scale was set using a known scale bar in each image. For each group, the following parameters were measured Cell Density, Collagen Density, Epidermal and Dermal Thickness, Vessel and Hair Follicle Density.

#### Statical analysis

Quantitative data are presented as mean ± standard deviation (SD). Statistical comparisons among groups were conducted using one-way analysis of variance (ANOVA), followed by Tukey’s post hoc test for multiple comparisons. Statistical significance was set at *p* < 0.05. All statistical analyses were performed using GraphPad Prism software (version 9.0; GraphPad Software, San Diego, CA, USA). To improve clarity and avoid overcrowding of the graphical presentation with multiple significance indicators. Cell density, collagen density, epidermal thickness, dermal thickness, vessel density, and hair follicle density were measured in regenerated skin on day 14 after wound.

## Results and discussion

Seven ternary AgVO_3_-HAP/GO@PCL nanocomposite samples were systematically prepared to comprehensively evaluate their wound-healing potential. Each sample was carefully designed to isolate and assess the individual contributions of AgVO_3_, HAp, and GO, along with their combined effects on the nanocomposite structure. This structured methodology allowed for an in-depth and comprehensive analysis of the interactions and performances of the composite components.

### Structural and microstructural properties

#### Synchrotron X-ray diffraction (SXRD)

**Fig. 2:**
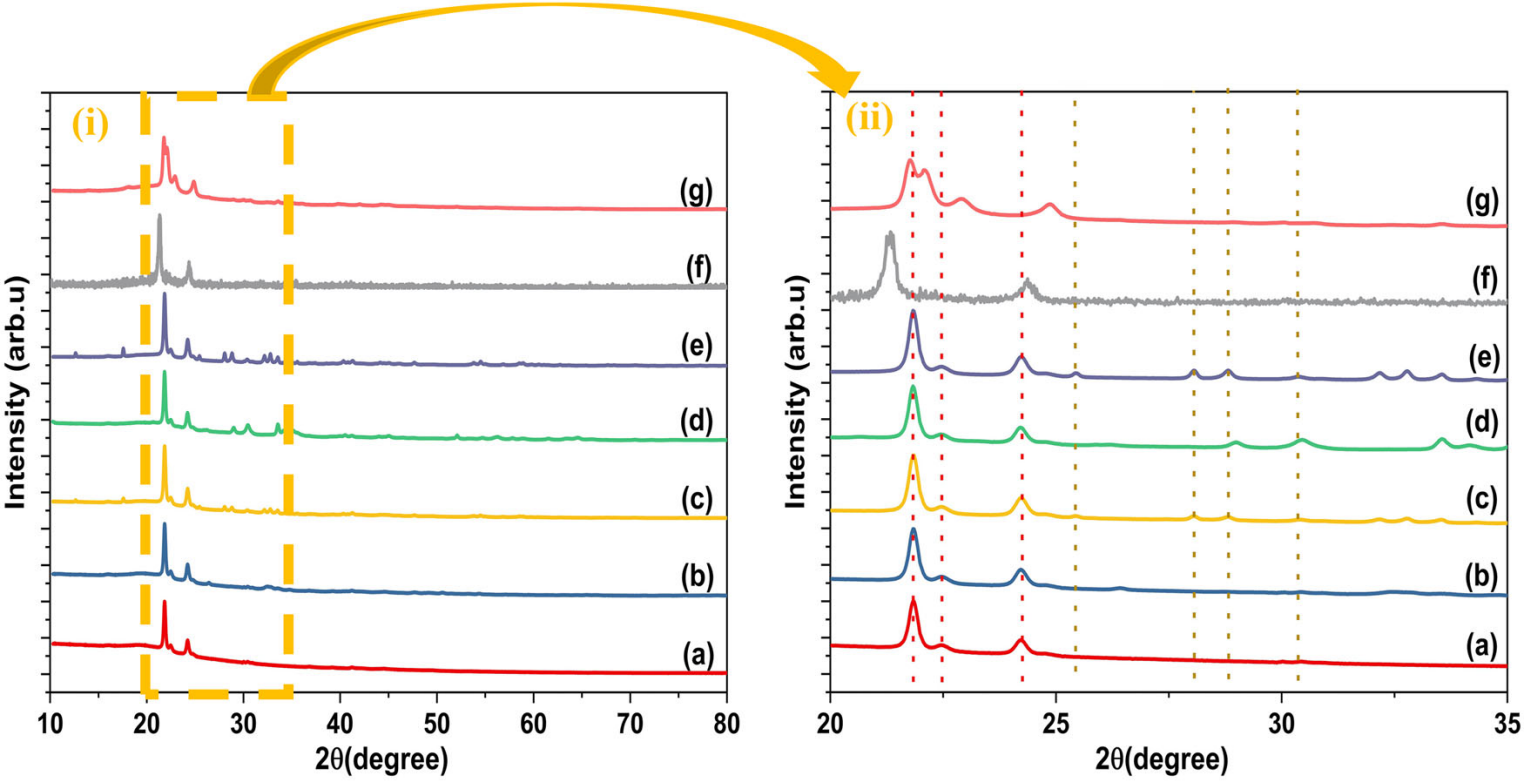
SXRD of **a** PCL, **b** HAP@PCL, **c** AgVO_3_@PCL, **d** AgVO_3_-HAP @PCL, **e** AgVO_3_/GO@PCL. **f** HAP/GO@PCL and **g** AgVO_3_-HAP/GO@PCL nanocomposites. (i) Full range of 2θ and (ii) magnified view of the 2θ region from 20° to 35°; vertical dashed lines indicate reference positions for characteristic diffraction peaks of each phase

The structural properties of the synthesized samples were examined using XRD, as depicted in Fig. 2. PCL is typically regarded as a semicrystalline polymer, featuring both ordered crystalline regions and disordered amorphous areas. In Fig. 2a, three distinct peaks are observed at 2θ = 21.80°, 22.47°, and 24.21°, corresponding to the (110), (111), and (200) planes of the orthorhombic crystal structure of PCL [32]. Variations in sample homogeneity can lead to differences in the intensity of the diffraction peaks [33]. Inhomogeneous samples may contain regions with different crystal orientations, resulting in varying intensities in the XRD patterns. According to the analysis, the crystallographic structure of HAp was identified as ICDD No. 00-064-0738 [34]. Additionally, Fig. 2c presents a typical XRD pattern of the synthesized AgVO_3_ nanostructure, prepared via a chemical precipitation method at room temperature. The data revealed the presence of both α- and β-AgVO_3_ phases, with no indication of other phases or silver residues, consistent with previously published data indexed by JCPDS cards. The diffraction peaks observed can be attributed to the β-AgVO_3_ phase (JCPDS 29-1154) [35], The main phase observed is the β-phase, along with a small presence of the α-AgVO_3_ phase (ICDD no. 01-089-4396) [35, 36], which shows relatively weak diffraction peaks compared to the β-phase. The primary peaks of HAp were observed at the (022), (121), (112), (030), (022), and (130) planes. Additionally, the characteristic peaks of AgVO_3_ were identified at 12.62°, 17.50°, 25.40°, 28.14°, 28.70°, 30.09°, 32.66°, and 33.71° (monoclinic C2/c), corresponding to the (110), (200), (221), (221), (221), (221), (-131), and (002) planes. It is noted that the metastable phase of AgVO_3_ was no longer present in the AgVO_3_-HAP composite, leaving only the β-phase. For the ternary composite, additional peaks appeared at 13.4°,which were attributed to the presence of reduced graphene oxide (rGO) [37].

Table 1 presents the crystallinity index (CI), crystallite size (D), and microstrain (ε) for all samples examined. The crystallinity index was determined using Eq. (1), employing the area method (deconvolution method). The high crystallinity index of pure PCL, found to be 79.63%, is also shown in Table 1. The slight shift in the peak positions is attributed to the differences in the molecular weights and crystal packing of the polymers. The crystallinity of PCL decreased with the addition of additives, except for the ternary composite (AgVO_3_-HAP/GO@PCL).

**Table 1:** The crystallinity index, crystallite size and microstrain for prepared samples

Sample	Crystallinity index (CI) (%)	Crystallite size (nm)	microstrain
PCL	79.63		
HAP@PCL	63.52	2.65$\times {10}^{2}$	0.213
AgVO_3_@PCL	62.15	2.02$\times {10}^{3}$	1.930
AgVO_3_-HAP@PCL	65.64	1.33$\times {10}^{3}$	0.709
AgVO_3_/GO@PCL	77.01	7.54$\times {10}^{2}$	0.213
HAP/GO@PCL	64.75	1.21$\times {10}^{2}$	0.107
AgVO_3_-HAP/GO@PCL	82.39	3.92$\times {10}^{2}$	0.414

The crystallite size and microstrain were calculated using the Williamson-Hall equation, as expressed in relation [2] and presented in Table 1.

*k* is the shape factor (typically 0.9 for spherical crystallites, such as HAP); however, for AgVO_3_ rod-shaped crystals with an aspect ratio (length to width) of approximately 10, it equals approximately 0.75. For fibrous crystals (such as nanofibers or microfibers), the shape factor *k* in the Scherrer equation is typically lower than that for spherical or equiaxed crystals. Owing to the high anisotropy of fibers with a large length-to-diameter ratio, the *k*-value generally decreases for highly elongated fibers (k ≈ 0.5 − 0.7) and moderately elongated fibers (k ≈ 0.7 − 0.9); however, 0.65 is commonly utilized [38].

As shown in Table 1 it is observed that the presence of AgVO_3_ increases the crystallite size, whereas GO and HAP decrease the crystallite size. This is because GO tends to fill the end-to-end chain of PCL, thereby decreasing the microstrain. The addition of HAP fills the remaining end-to-end chain spaces, whereas AgVO_3_ occupies the spaces between the polymer chains.

#### Fourier transformation infrared spectroscopy (FTIR)

**Fig. 3:**
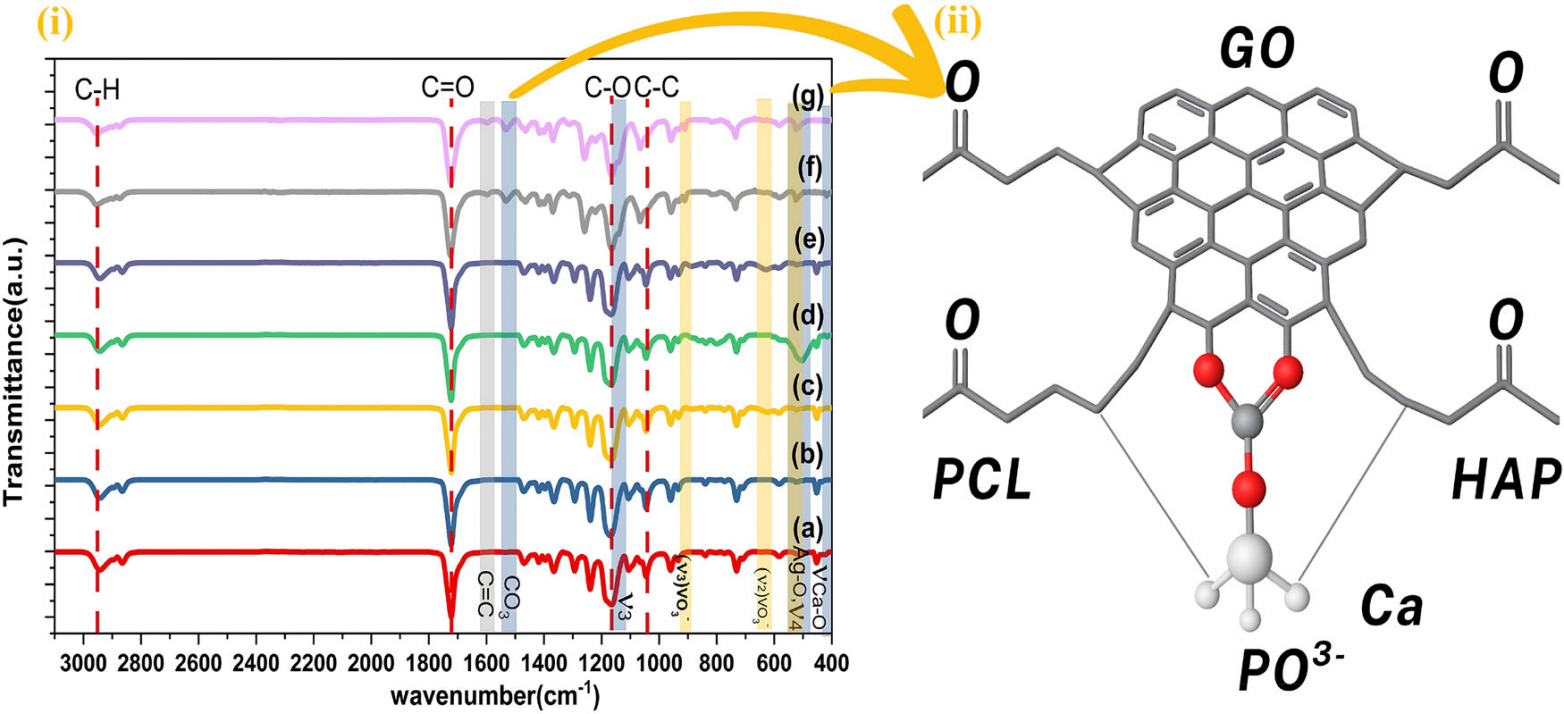
i FTIR spectra of **a** PCL, **b** HAP@PCL, **c** AgVO_3_@PCL, **d** AgVO_3_-HAP @PCL, **e** AgVO_3_/GO@PCL. **f** HAP/GO@PCL, and **g** AgVO_3_-HAP/GO@PCL. (ii) The schematic illustration highlights the molecular interaction between PCL chains, GO sheets, and HAP

To assess the incorporation of GO and HAP into the end to end of the PCL chain, FTIR analysis of the composites was performed, as shown in Fig. 3i The intensity of this band decreased with the inclusion of GO and HAP, implying the interaction of GO and PO_4_^3−^ of HAP with C at the end of the PCL chain, forming the stretching band of the carbonate group at 1500 cm^−1^ [39]. The schematic illustration that highlights the molecular interaction between PCL chains, GO sheets and Hap is shown in Fig. 3ii. The presence of carbonate significantly impacts the chemical and thermal stability of HAP [40]. FTIR analysis revealed bands that confirmed the chemical composition of both the powder and the fibrous ternary composite of the AgVO_3_-HAP/GO components. The precise positions and intensities of these peaks can vary depending on factors such as the molecular weight, crystallinity, and processing conditions of the polymer [41]. The FTIR spectra of the synthesized samples are presented in Fig. 3. The peaks around 2945 cm^−1^ were attributed to the, while a strong peak at approximately 1724 cm^−1^ corresponded to the carbonyl group (C = O) stretching in the PCL backbone [42]. The peak near 1126 cm^−1^ reflects the stretching vibrations of the ester groups (C-O-C) in the polymer chain [43], and the peaks around 1040 cm^−1^ are linked to the stretching vibrations of the carbon-carbon (C-C) bonds in the polymer backbone [44]. Bending vibrations of methylene (CH₂) groups in the polymer chain appear at approximately 1420–1470 cm^−1^ [45]. Furthermore, bands corresponding to HAP and AgVO_3_ were observed. The bands at 550–569 cm^−1^ correspond to the (ν_4_) phosphate rocking vibrations [46]. Strong absorption bands in the range of 1118–1150 cm^−1^ represent the ν_3_ stretching vibrations of the phosphate group [47]. arising from the symmetric and asymmetric stretching of the PO₄^3 −^ ions. An Ag-O stretching vibration band appears between 506 and 540 cm^−1^ [48]. The vanadate bending vibrations (ν_2_) and stretching vibrations (ν_3_) of the VO₃^−^ group were observed in the 604–660 cm^−1^ and 900–930 cm^−1^ regions, respectively [49]. An absorption peak related to sp^2^ carbon-carbon (C = C) bonds in the graphene lattice is observed at approximately 1600 cm^−1^ [50], although these peaks may be diminished or shifted due to the presence of oxygen-containing functional groups.

**Fig. 4:**
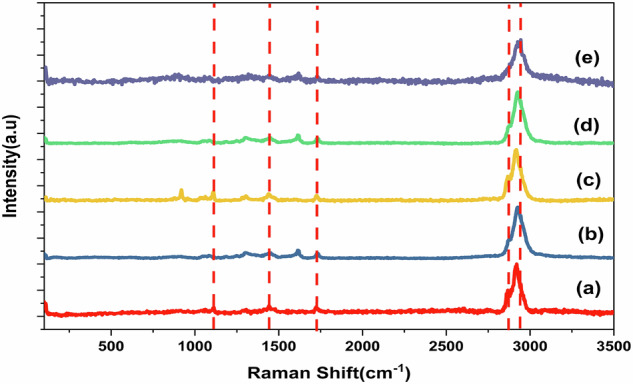
Raman spectra of **a** PCL, **b** AgVO_3_-HAP @PCL, and **c** AgVO_3_/GO@PCL. **d** HAP/GO@PCL, and **e** AgVO_3_-HAP/GO@PCL

Raman spectroscopy is a non-destructive method for examining the structural and chemical properties of GO, providing insights into its oxidation state, functional groups, and vibrational modes. A key feature of the GO spectrum is the G-band, which appears at approximately 1580 cm-1, corresponding to the in-plane stretching vibrations of sp^2^ carbon atoms, indicating a graphitic structure. The spectra were recorded at room temperature, as illustrated in Fig. 4c–e [51]. The D-band, located at approximately 1350 cm^−1^, represents the breathing mode of κ-point phonons with A_1_g symmetry and serves as an indicator of structural defects, such as vacancies, edges, and sp^3^-hybridized carbon atoms, which arise during the oxidation process [52, 53]. Another significant feature in the Raman spectrum is the 2D-band, which appears in the 2700–2900 cm^−1^ range. As a second-order overtone of the D-band, it provides valuable insights into the number of graphene layers and the degree of structural disorder in GO [54]. Essential spectral parameters, including the intensity ratio of the D-band to the G-band (ID/IG) and the full width at half maximum (FWHM) of the 2D-band, are critical for assessing the structural characteristics and functional properties of GO in nanocomposite scaffolds [55]. These parameters are crucial for elucidating the structural properties and functional behavior of GO in nanocomposite scaffolds. The parameters outlined in Table 2 are frequently employed to assess the level of disorder and dimensions of the sp^2^ domains in the GO structure.

**Table 2:** Raman-derived structural parameters of GO-based electrospun scaffolds, including crystallite size (L_a_), average defect distance (L_D_), defect density (n_D_), and in-plane sp^2^ domain size (L_SP_^2^)

Samples	I_D_/I_G_	L_a_(nm)	L_D_ (nm)	n_D_ (cm^−2^)	L_sp2_(nm)
AgVO_3_/GO@PCL	0.63	30.42	15.19	1.36*10^11^	30.06
HAP/GO@PCL	1.37	14.03	10.32	2.95*10^11^	13.86
AgVO_3_-HAP/GO@PCL	0.67	28.82	14.79	1.44*10^11^	28.49

The values were calculated using the intensity ratio of the D and G bands (I_D_/I_G_) in the Raman spectra

The PCL baseline exhibited peaks at 2950, 2915, and 2871 cm^−1^, attributed to C–H stretching [56], while the peak at 1726 cm^−1^ corresponded to C = O stretching [57]. Peaks at 1441, 1109, and 564 cm^−1^ are associated with CH₂ bending, C–O–C stretching [58], and C–C skeletal bending [59], respectively, as shown in Fig. 4a.

The incorporation of HAP-AgVO_3_ into PCL resulted in shifts at 3006 cm^−1^ and 2930 cm^−1^ in the C–H stretching, indicating alterations in the polymer matrix due to interactions with HAP and AgVO_3_. The peaks at 850 cm^−1^, 977 cm^−1^, and 920 cm^−1^ are attributed to PO_4_^3−^symmetric stretching from HAP [60], V = O stretching modes of AgVO_3_, and potential overlap with phosphate (PO_4_^3−^) vibrations from HAP, respectively. A slight shift to 1730 cm^−1^ from the PCL C = O stretching suggests chemical bonding with (PO_4_^3−^) of HAP, which is confirmed by FTIR analysis. Peaks at 404, 359, and 470 cm^−1^ are associated with the characteristic bending mode peaks of Ag–O or V–O bending modes detected at 404, 309, and 470 cm^−1^ [61, 62], respectively.

The addition of AgVO_3_ and GO to PCL, as shown in Fig. 4c, induces shifts at 2919 and 2863 cm^−1^ in the C–H stretching, indicating structural modifications in the polymer. The peaks at 1601 and 1542 cm^−1^ were assigned to the G and D bands of GO, revealing defects and oxidation-related bonding. The peak at 1369 cm^−1^ is attributed to the D band of GO, signifying structural disorder. The peaks at 1109 and 1070 cm^−1^ likely represent C–O–C stretching in PCL overlapping with the V–O stretching of AgVO_3_. Peaks at 471 and 520 cm^−1^ indicate V–O–V bending or lattice vibrations from AgVO_3_ [63].

In the ternary composite (AgVO_3_, HAP, and GO added to the PCL mat), as shown in Fig. 4e, significant shifts at 3009, 2931, and 2863 cm^−1^ in C–H stretching suggest enhanced interaction within the composite. The peak at 2715 cm^−1^ potentially represents the 2D band of GO, indicating reduced graphene layers [64]. The peak at 1600 cm^−1^ corresponds to the G band of GO and indicates potential interaction with AgVO_3_. A slightly shifted peak at 1731 cm^−1^ for C = O stretching indicates a strong interaction [65] with both HAP and GO [66]. The peaks at 878 and 893 cm^−1^ represent V–O terminal stretching from AgVO_3_ and possible overlap with phosphate vibrations, respectively. The peaks at 440 and 96 cm^−1^ are associated with the low-frequency AgVO_3_ lattice modes. From Table 2 The data demonstrate that the incorporation of AgVO_3_ and the ternary combination of AgVO_3_-HAP/GO@PCL reduces defects and enhances the structural quality of the scaffolds compared to HAP/GO@PCL alone. These insights are critical for understanding the performance of materials in biomedical applications.

#### Morphological features

**Fig. 5:**
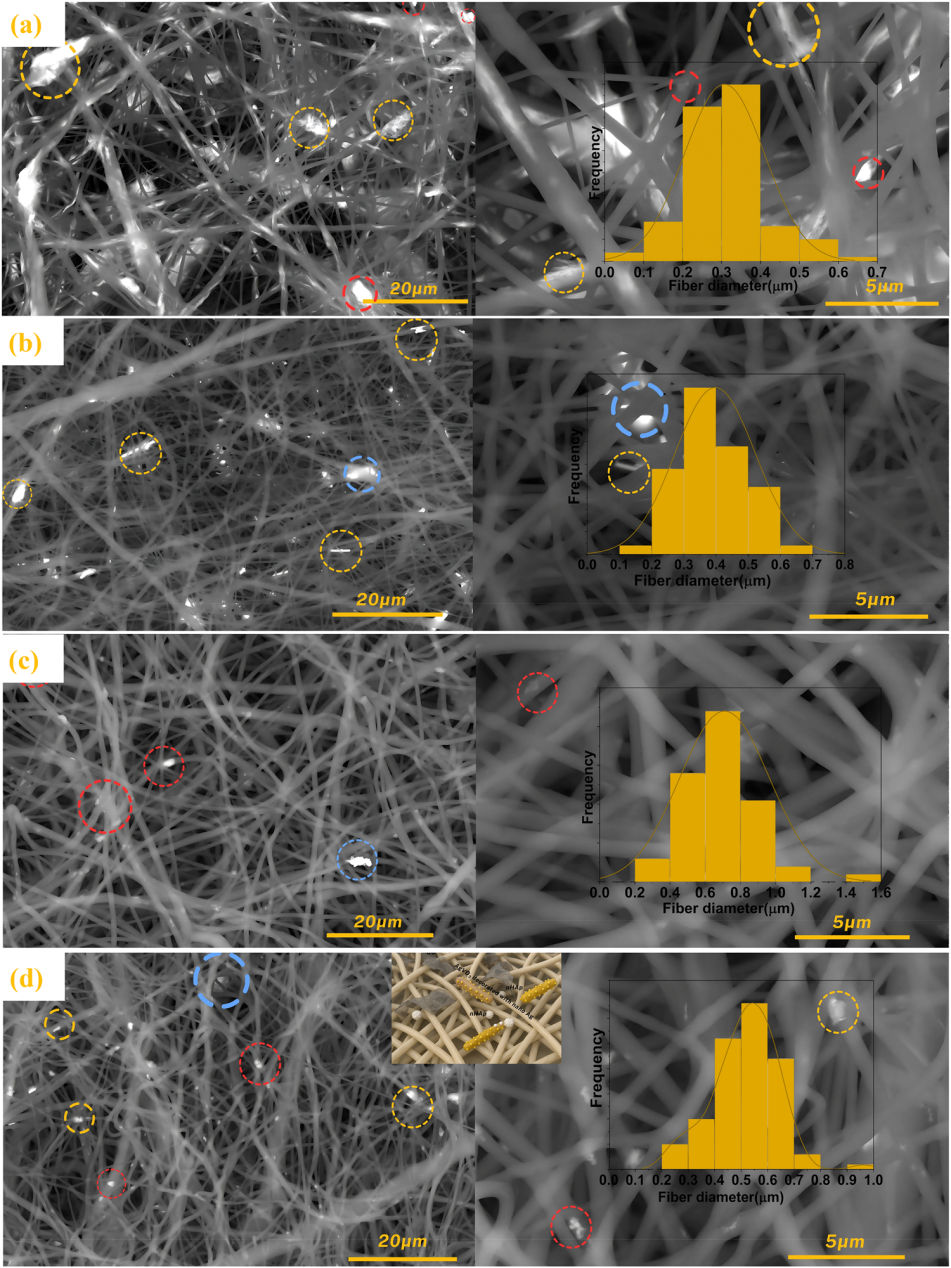
FE-SEM micrographs of **a** AgVO_3_-HAP @PCL, **b** AgVO_3_/GO@PCL, **c** HAP/GO@PCL, and **d** AgVO_3_-HAP/GO@PCL (inset is a 3D schematic model of the composit). yellow dashed circles show AgVO_3_ rods, red dashed circles show nHAp and blue dashed circles show GO. A fiber diameter histogram is inserted for each scaffold

The SEM micrographs of the samples, shown in Fig. 5, confirmed the absence of bead morphology in all porous scaffolds, indicating the formation of high-quality, randomly oriented, branched, and straight fibers. The fiber morphology and diameter varied significantly depending on the additives incorporated into scaffolds. The inclusion of AgVO_3_-HAP led to pronounced changes in the fiber morphology, as shown in Fig. 5a. The fibers exhibited a reflective surface with increased intertwining and enhanced random orientation. The average fiber diameter decreased to 0.3 µm, accompanied by an increase in the number and size of pores.

**Fig. 6:**
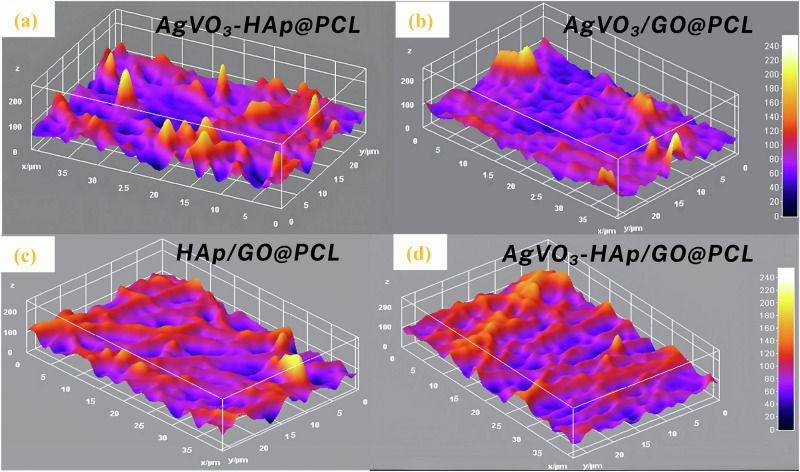
3D FE-SEM topography images of electrospun scaffolds: **a** AgVO_3_-HAP@PCL, **b** AgVO_3_/GO@PCL, **c** HAP/GO@PCL, and **d** AgVO_3_-HAP/GO@PCL

For AgVO_3_/GO@PCL scaffolds, shown in Fig. 5b, had fibers randomly oriented in all directions, with an average diameter of 0.35 µm. The number of pores increased, whereas their size decreased significantly. AgVO_3_ was distributed non-uniformly across the fiber surface. The average surface roughness decreased to 47.9 nm, indicating an elevated surface energy that facilitates interactions with the surrounding medium, as shown in Fig. 6b. This observation aligns with the contact angle measurements, which recorded an angle of 75.85 °, suggesting improved hydrophilicity. The HAP/GO@PCL scaffolds displayed a fiber morphology in which graphene-like structures appeared to wrap around the fibers, as shown in Fig. 5c. This configuration increased the average fiber diameter to 0.7 µm. When AgVO_3_ was added to HAP/GO, AgVO_3_-HAP/GO@PCL scaffolds were formed (Fig. 5d), and the fiber diameter decreased to 0.55 µm. The micrographs revealed that GO contributed to the enlargement of the fiber diameters and altered the fiber structure by encapsulating the fibers. The average surface roughness of HAP/GO@PCL was 54 nm, which increased to 64 nm for AgVO_3_-HAP/GO@PCL scaffolds, confirming the data and explanation XRD and FTIR data. GO tends to fill the end-to-end chain spaces of the polymer, whereas AgVO_3_ decorates the surface of the fiber. This suggests that incorporating graphene oxide reduces the surface roughness while enhancing the scaffold functionality. Table 3 summarizes the surface roughness parameters of the different fiber samples, providing a comprehensive comparison based on the key metrics.

**Table 3:** Surface Roughness Characteristics of Composite Fiber Samples Average Roughness (R_a_), Root Mean Square Roughness (R_q_), Maximum Roughness Height (R_t_), Maximum Valley Depth (R_v_) and Maximum Peak Height (R_p_)

Sample	Fiber diameter (µm)	R_a_ (µm)	R_q_ (µm)	R_t_ (µm)	R_v_ (µm)	R_p_ (µm)
AgVO_3_-HAP@PCL	0.30	7.13	0.097	0.871	0.347	0.524
AgVO_3_/GO@PCL	0.40	0.048	0.719	0.805	0.309	0.496
HAP/GO@PCL	0.70	0.054	0.069	0.516	0.223	0.293
AgVO_3_-HAP/GO@PCL	0.55	0.064	0.084	0.817	0.413	0.404

Figure 6 shows the surface roughness analysis of the composite fiber scaffolds, which revealed significant variations in the topographical features depending on the incorporated components. Among the samples, AgVO_3_-HAP@PCL exhibited the highest average roughness (R_a_= 7.13 µm), indicating a highly textured surface that may enhance cell adhesion. In contrast, AgVO_3_/GO@PCL and HAP/GO@PCL displayed much lower R_a_ values (0.048 µm and 0.054 µm, respectively), reflecting smoother surfaces, likely due to the planar structure of GO facilitating better fiber alignment. The maximum roughness height (R_t_) and maximum peak height (R_p_) followed similar trends, with AgVO_3_-HAP@PCL exhibiting the most pronounced peaks and valleys. Interestingly, although AgVO_3_-HAP@PCL and AgVO_3_/GO@PCL had comparable R_t_ values (0.871 and 0.805 µm, respectively) as shown in Table 3, the former demonstrated a higher R_p_, suggesting sharper protrusions. These surface characteristics may influence the wettability and biological response of the scaffolds, with rougher textures generally supporting better cellular interactions in tissue-engineering applications.

### Mechanical properties

**Fig. 7:**
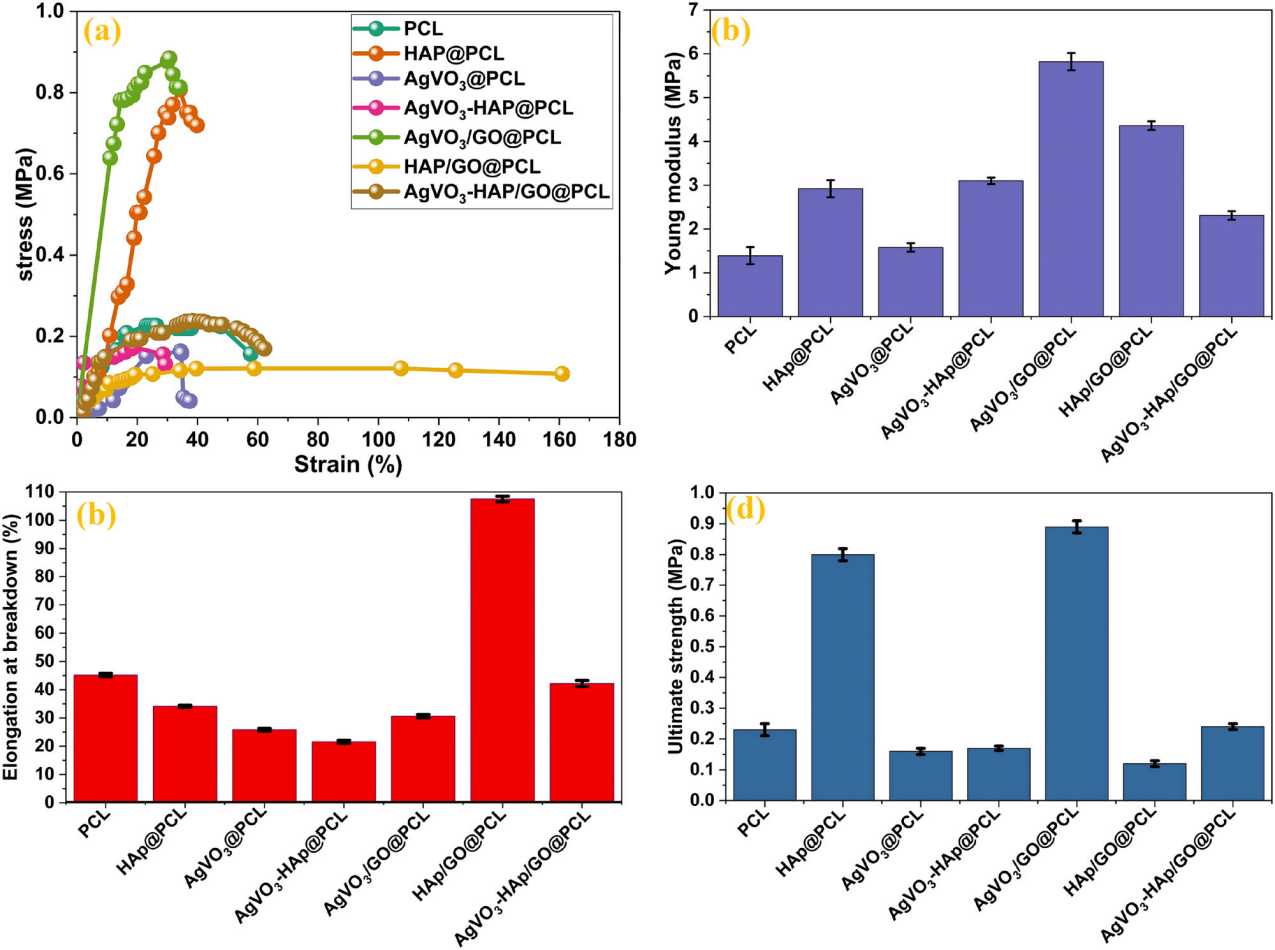
Mechanical properties of the electrospun scaffolds. **a** Stress-strain curve, **b** Young’s modulus, **c** Elongation at breakdown (%) and **d** Ultimate strength (MPa) of the prepared samples of PCL, HAP@PCL, AgVO_3_@PCL, AgVO_3_-HAP @PCL, AgVO_3_/GO@PCL, HAP/GO@PCL, and AgVO_3_-HAP/GO@PCL

PCL is widely recognized for its excellent biocompatibility, biodegradability, and mechanical properties that closely mimic those of natural tissues. One of its key advantages is its slow degradation rate in vivo, making it an ideal material for tissue engineering and wound healing applications. To enhance the mechanical strength of PCL scaffolds, reinforcement materials such as HAP, GO, and AgVO_3_ were incorporated. These reinforcements significantly improved the structural integrity of PCL, optimizing it for specialized biomedical applications. In this study, the stress-strain characteristics of the prepared composite samples were evaluated, as illustrated in Fig. 7a. From these evaluations, critical mechanical parameters, including the ultimate tensile strength, elongation at break, and Young’s modulus, were determined and are summarized in Table 4. These parameters are pivotal for designing scaffolds that can withstand the mechanical demands of tissue engineering and wound-dressing applications, ensuring durability and functionality.

**Table 4:** Mechanical properties of electrospun composite scaffolds, including ultimate tensile strength (KJ/m^3^), elongation at break (%), and Young’s modulus (MPa)

Sample	Ultimate strength (KJ/m^3^)	Elongation breakdown (%)	Modulus (MPa)
PCL	0.23	45.18	1.39
HAP@PCL	0.80	34.12	2.92
AgVO_3_@PCL	0.16	25.83	1.57
AgVO_3_-HAP@PCL	0.17	21.59	3.10
AgVO_3_/GO@PCL	0.88	30.64	5.82
HAP/GO@PCL	0.12	107.54	4.36
AgVO_3_-HAP/GO@PCL	0.24	42.20	2.31

The ultimate strength of PCL increased substantially with the addition of HAP and GO, with AgVO_3_/GO@PCL exhibiting the highest value (0.88 kJ/m^3^), as revealed in Fig. 7d. This improvement is attributed to the combined effects of AgVO_3_’s monoclinic crystal structure and GO’s exceptional tensile strength and modulus of GO. The Young’s modulus also showed a notable enhancement in the composites, particularly in AgVO_3_-HAP@PCL (3.10 MPa), indicating increased stiffness and the most rigid, thereby enhancing the polymer elasticity, as shown in Fig. 7b. The bioceramic material HAP, known for its high hardness, played a significant role in this enhancement, particularly in HAP@PCL composites. The elasticity of the nanofibers, measured by the elongation at break, was highest for HAP/GO@PCL (107.54%), indicating excellent flexibility and elongation capacity, as shown in Fig. 7c. However, the elongation decreased in AgVO_3_-HAP@PCL, suggesting a trade-off between enhanced stiffness and reduced elasticity. Toughness, defined as the energy required to break the scaffold, reached its maximum value for AgVO_3_/GO@PCL (0.88 kJ/m^3^). This enhancement is likely due to the impermeability of GO and the structural reinforcement provided by rod-shaped AgVO_3_ particles.

The incorporation of reinforcement materials allows PCL scaffolds to be tailored to the specific mechanical requirements of biomedical applications [67]. HAP enhances tensile strength and modulus, making it suitable for load-bearing applications. GO improves the toughness and barrier properties, making it ideal for packaging and membrane applications [68]. AgVO_3_, with its monoclinic structure, enhances tensile strength and modulus, making it suitable for wound dressings that require high mechanical resilience [69]. These findings highlight that the combination of materials leads to the creation of PCL-based scaffolds with enhanced mechanical properties, thereby broadening their potential for various biomedical applications. Compared with traditional wound dressings, such as cotton gauze, hydrogels, or paraffin-based pads, which often exhibit poor mechanical strength, limited elasticity, and low structural integrity, electrospun PCL-based composite scaffolds demonstrate substantially improved mechanical performance. Conventional dressings typically lack the tensile strength and elasticity required to withstand movement and stress at the wound site, leading to poor adhesion, wrinkling, and early degradation.

To contextualize the mechanical performance of the developed scaffolds, it is essential to compare them with conventional wound-dressing materials. Typical wound dressings based on natural or synthetic polymers, such as collagen, chitosan, and electrospun polycaprolactone (PCL), demonstrate ultimate tensile strengths ranging from 0.1 to 5 MPa, Young’s modulus values between 0.5 and 20 MPa, depending on flexibility requirements, and elongation at break values exceeding 50%, particularly for skin-contact applications. For instance, the commercial hydrocolloid dressing DuoDERM Extra Thin exhibits an elongation at break of approximately 71.4% [70], whereas our HAP/GO@PCL scaffold achieved a significantly higher value of 107.54%, indicating superior flexibility and stretchability.

Furthermore, the AgVO_3_-HAP@PCL scaffold demonstrated a Young’s modulus of 6.26 MPa, which exceeds that of Sol/SPI/Mp nanofibers previously reported at 3.6 MPa [71]. This highlights the enhanced stiffness of our composite structure. Additionally, the AgVO_3_-HAP/GO@PCL scaffold exhibited a Young’s modulus of 2.31 MPa, surpassing that of electrospun gentamicin-loaded pullulan/PVA/gum arabic nanofibers, which ranged between 1.5 and 2 MPa [72]. These comparisons underscore the mechanical robustness of the fabricated scaffolds, positioning them as competitive and potentially superior candidates for next-generation wound-dressing applications.

### Contact angle measurements

An optimal wound dressing must balance moisture retention and management to support healing while preventing the maceration of the skin. The contact angle of a material is a critical factor that influences fluid absorption, skin adhesion, and microbial resistance [73]. Hydrophilic dressings with low contact angles efficiently absorb exudate, reduce adherence to the wound, and enhance patient comfort during removal [74, 75]. In contrast, hydrophobic materials may hinder fluid handling and impair healing processes. Furthermore, surface wettability affects microbial adhesion, making the contact angle a key parameter in designing dressings that promote tissue regeneration and minimize the risk of infection. A material with an appropriate contact angle can conform securely to the wound site without causing trauma upon removal [76].

**Fig. 8:**
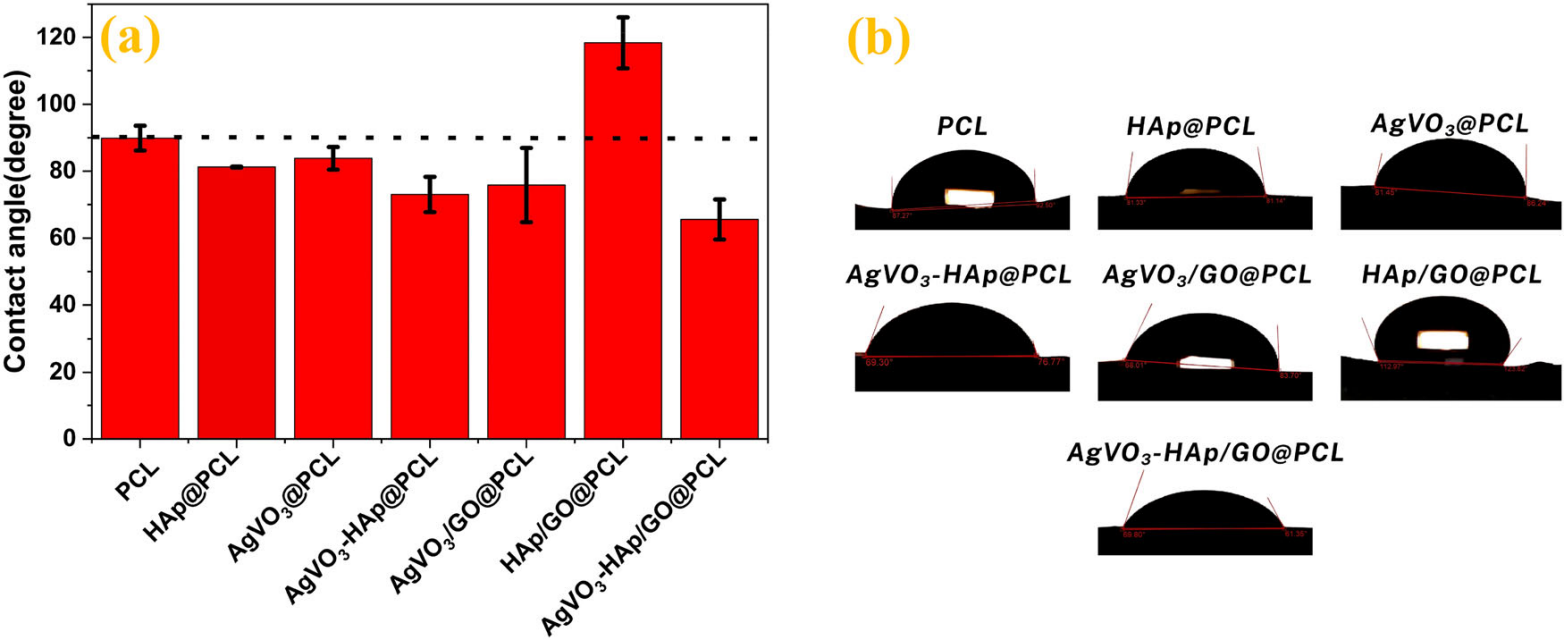
Static water contact angle measurements of the fabricated scaffolds. **a** Bar graph showing the average contact angle (in degrees) for PCL, HAP@PCL, AgVO_3_@PCL, AgVO_3_-HAP@PCL, AgVO_3_/GO@PCL, HAP/GO@PCL, and AgVO_3_-HAP/GO@PCL. The dashed line at 90° indicates the threshold between hydrophilic ( < 90°) and hydrophobic ( > 90°) behaviors. **b** Representative contact angle images of water droplets on each scaffold surface

The wettability of the prepared scaffolds was assessed based on the contact angle measurements shown in Fig. 8a, b. The AgVO_3_-HAP/GO@PCL scaffold exhibited a contact angle of 65.6°, as depicted in Table 5, indicating moderate hydrophilicity. This value suggests that the scaffold effectively absorbs wound exudate while maintaining an optimal moisture balance, which is crucial for preventing maceration and promoting healing of the wound. In contrast, the neat PCL scaffold exhibited a contact angle of 89.89 ± 3.79°as depicted in Table 5, indicating moderate hydrophilicity but a higher tendency to repel moisture. However, the HAP/GO@PCL scaffold displayed hydrophobic behavior with a contact angle of 118.40 ± 7.67°, which could limit its ability to interact with the wound exudate, confirming that GO sheets wrapped around the PCL fiber, as depicted in the SEM micrographs. The contact angle of AgVO_3_/GO@PCL was 75.85°, suggesting increased hydrophilicity compared to pure PCL. This increased wettability is likely linked to enhanced antibacterial activity, as the surface energy of the scaffold facilitates better interaction with the surrounding medium, promoting moisture absorption, and inhibiting microbial growth. The unique properties of GO, with its combination of hydrophilic functional groups (such as hydroxyl, epoxy, and carboxyl) and hydrophobic graphene regions, contribute to the complex surface behavior of GO@PCL composites. The aggregation of GO particles on the PCL matrix may alter the roughness of the surface, exposing more hydrophobic regions of the graphene structure while shielding hydrophilic sites, which can impact the overall wettability and moisture management of the scaffold. These findings indicate that the novel AgVO_3_-HAP/GO@PCL scaffolds possess optimal hydrophilicity and surface properties, promoting effective wound healing by maintaining a balanced moisture environment, enhancing antibacterial activity, and supporting tissue regeneration.

**Table 5:** The Static water contact angle measurements of various electrospun scaffolds

Sample	Contact angle	Ref
PCL	89.89 ± 3.70	[80, 81]
HAP@PCL	81 ± 0.13	[82]
AgVO_3_@PCL	83.85 ± 3.39	__
AgVO_3_-HAP@PCL	73.04 ± 5.28	__
AgVO_3_/GO@PCL	75.86 ± 11.09	__
HAP/GO@PCL	118.40 ± 7.67	__
AgVO_3_-HAP/GO@PCL	65.58 ± 5.97	__

### Antibacterial activity

Bacterial infections are a major cause of delayed or failed tissue healing after injury. To address this challenge, it is crucial to incorporate antibacterial properties into scaffold biomaterials to prevent infection and support tissue integration. Ideally, these antibacterial agents should be gradually released into the wound environment to avoid burst release, which can lead to toxicity.

**Fig. 9:**
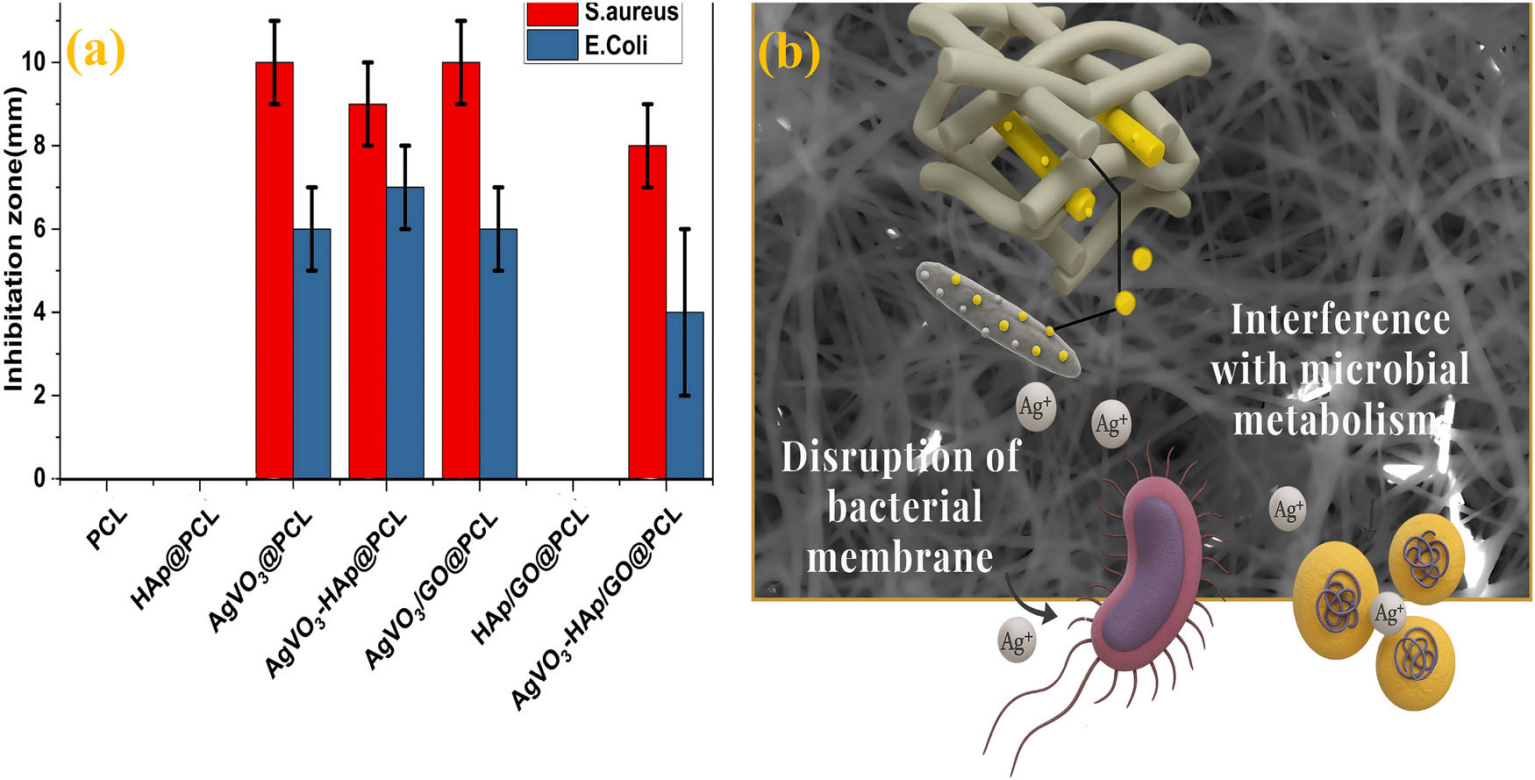
Antibacterial performance of electrospun scaffolds against *S. aureus* and *E. coli*. **a** Inhibition zone diameters for PCL, HAP@PCL, AgVO_3_@PCL, AgVO_3_-HAP @PCL, AgVO_3_/GO@PCL, HAP/GO@PCL, and AgVO_3_-HAP/GO@PCL. **b** Schematic representation of the antibacterial mechanism, illustrating AgVO_3_ nanorods embedded in the PCL matrix, releasing Ag⁺ ions

In this study, AgVO_3_ was chosen as an additive for PCL scaffolds because of its recognized antibacterial properties, which aid in preventing bacterial growth, reducing inflammation, and promoting wound healing. Antibacterial testing was conducted against both Gram-negative *E. coli* and gram-positive bacteria. The control samples, which consisted of untreated agar plates inoculated with bacteria, exhibited no inhibition zone. Seven different scaffolds were evaluated for antibacterial activity, and four of these samples demonstrated notable inhibition zones, as shown in Fig. 9a. The inhibition zones ranged from 8 to 10 mm for the most effective samples against S. aureus and from 4 to 7 mm against E. coli. Importantly, all scaffolds exhibiting antibacterial activity contained AgVO_3_, highlighting its critical role in promoting antibacterial properties.

The AgVO_3_@PCL and AgVO_3_/GO@PCL samples exhibited the strongest antibacterial activity. The antibacterial mechanism of Ag ions is not fully understood but is believed to involve their interaction with bacterial cell walls, leading to the generation of reactive oxygen species (ROS). Additionally, Ag ions can bind to intracellular bacterial enzymes, causing DNA damage and disrupting bacterial metabolism, ultimately resulting in the collapse of bacterial cells, as shown in Fig. 9b. The incorporation of AgVO_3_ into PCL scaffolds provides dual benefits: promoting wound healing and offering antibacterial protection. The combination of healing promotion and antibacterial efficacy makes these nanocomposite scaffolds promising candidates for clinical use in wound management.

### Wound healing

**Fig. 10:**
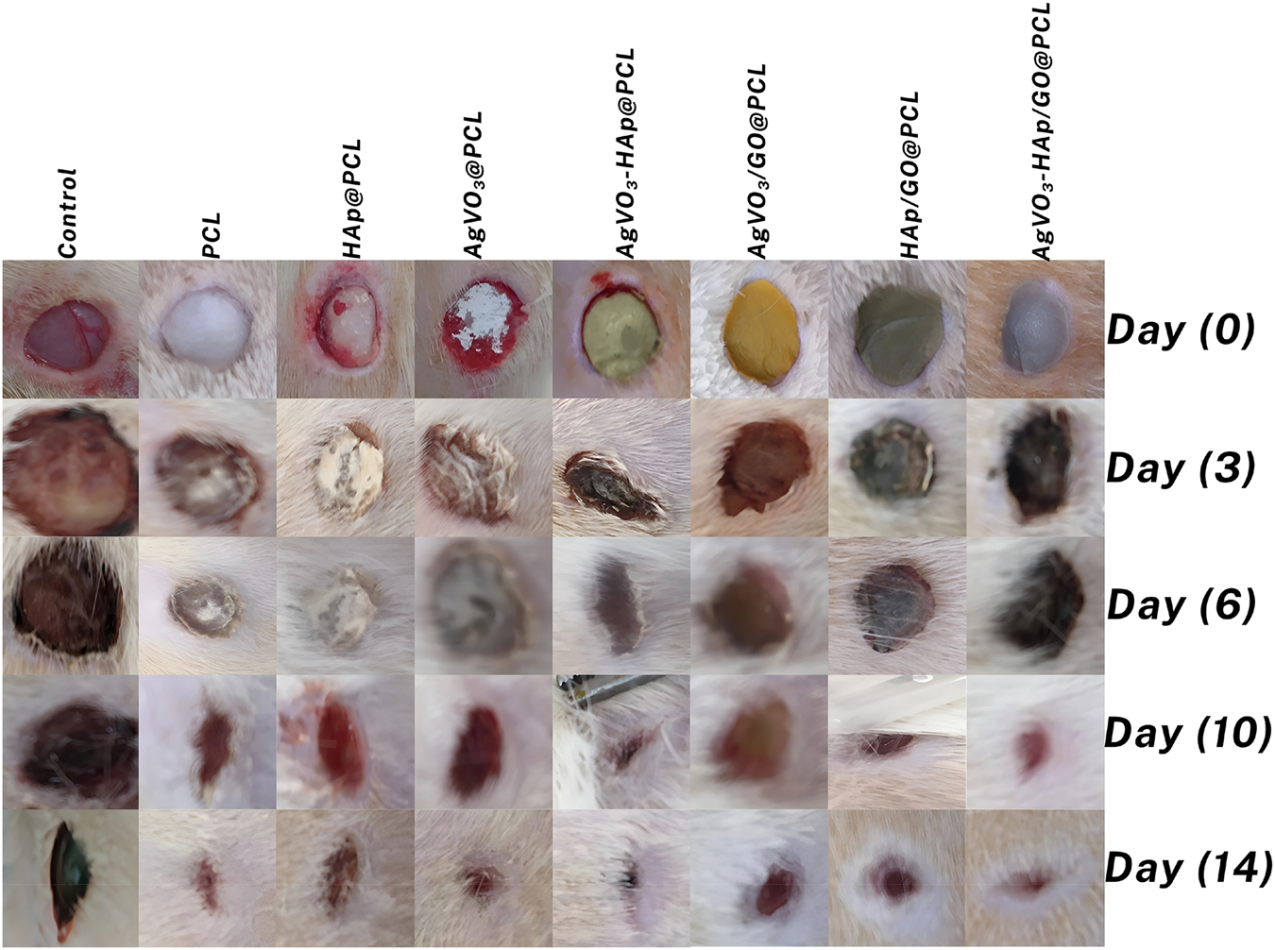
Macroscopic evaluation of wound healing over 14 days in the different treatment groups. Images were captured at Days 0, 3, 6, 10, and 14 for PCL, HAP@PCL, AgVO_3_@PCL, AgVO_3_-HAP @PCL, AgVO_3_/GO@PCL, HAP/GO@PCL, and AgVO_3_-HAP/GO@PCL

**Fig. 11:**
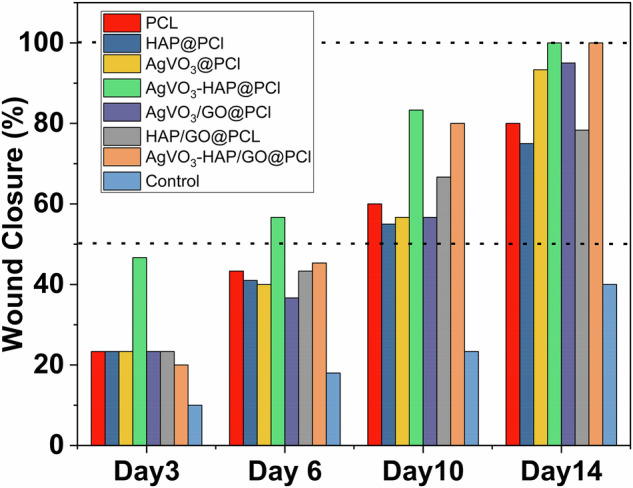
Wound closure at day 3,6, 10 and 14 for PCL, HAP@PCL, AgVO_3_@PCL, AgVO_3_-HAP @PCL, AgVO_3_/GO@PCL, HAP/GO@PCL, and AgVO_3_-HAP/GO@PCL

To address these challenges, advanced nanofibrous scaffolds have been developed and evaluated for their ability to accelerate wound healing processes. The AgVO_3_-HAP/GO@PCL scaffolds were designed to offer antimicrobial protection, support tissue regeneration, and provide a suitable microenvironment for cell proliferation and migration. The healing process as showed in Fig. 10 was monitored through optical imaging at key time points (days 0, 3, 6, 10, and 14) after treatment with various scaffolds. The untreated control group showed minimal healing progress, highlighting the need for effective wound management. The wound area was measured and normalized to the initial wound size to determine the healing rate for the different treatments. Figure 10 illustrates the wound healing process. By day 3, the AgVO_3_-HAP@PCL scaffolds achieved the highest average healing rate of 50%, with wounds reduced to half their original size compared to the control as presented in Fig. 11. By day 10, wounds treated with AgVO_3_-HAP@PCL and AgVO_3_-HAP/GO@PCL scaffolds had shrunk to one-fifth of their original size, significantly outperforming the other groups. At the end of the 14-day study, wounds treated with these scaffolds showed complete closure with no visible scarring or edge inflammation. The difference in healing rates between the treatment groups was significant (*p* < 0.05). Scaffolds incorporating GO have demonstrated superior mechanical properties and enhanced antibacterial effects, further improving their performance in wound closure and tissue regeneration. GO helps in modifying the surface morphology and architecture of the fiber and increases the active surface, which aids in wound healing. These results underscore the potential of AgVO_3_-HAP/GO@PCL scaffolds as effective wound dressings, facilitating rapid healing and preventing complications such as infection and excessive inflammation. Their novel composition allows for a combination of antimicrobial properties (from AgVO_3_), structural support (from HAP), and enhanced cell proliferation (from rGO). This study demonstrates the feasibility of these scaffolds as advanced wound care solutions, offering significant advantages over traditional dressings, particularly for chronic and infected wounds.

Effective wound healing is often compromised by factors such as bacterial or fungal contamination, chronic inflammation, mechanical stress, and inadequate wound care. Infections not only impair cellular functions but also prolong inflammation, delaying tissue repair and regeneration. While inflammation is a critical component of the initial wound healing process, excessive or persistent inflammation, common in chronic conditions such as diabetes, obesity, hypertension, or autoimmune disorders, can disrupt normal physiological pathways and compromise tissue integrity. Moreover, improper wound management, including poorly designed dressings, can exacerbate mechanical stress, impede healing, and lead to complications such as hypertrophic scarring or wound dehiscence [77, 78].

Table 6 highlights the superior performance of the AgVO_3_/GO@PCL and AgVO_3_-HAP/GO@PCL scaffolds in wound healing compared to other materials. While Silk Fibroin-based and Soluplus-SPI dressers achieved 90% closure on day 14, our composites demonstrated faster healing (50% by day 3) and complete closure by day 14. This superiority is attributed to the synergistic effects of the antibacterial (AgVO_3_), conductive (GO), and osteoinductive (HAP) components, combined with PCL’s structural support, promoting rapid re-epithelialization, reduced inflammation, and effective tissue remodeling.

**Table 6:** comparison of the wound healing efficacy of various wound dressings

Wound dresser	Results	Ref.
Silk Fibroin with both Hyaluronic Acid and Sodium Alginate	Achieved 90% wound closure after 14 days.	[83]
Soluplus-soy protein isolate (Sol-SPI) containing mupirocin (Mp) nanofiber	Achieved 90% wound closure after 14 days.	[71]
The biopolymer blends of SF and polysaccharides with HAP	Achieved 40% wound closure after 3 days.	[84]
Our work	AgVO_3_/GO@PCL achieved 50% wound closure after 3 days, and AgVO_3_/GO@PCL and AgVO_3_-HAP/GO@PCL achieved complete closure after 14 days.	

### Histological analysis of skin tissue sections

#### Histological assessment

**Fig. 12:**
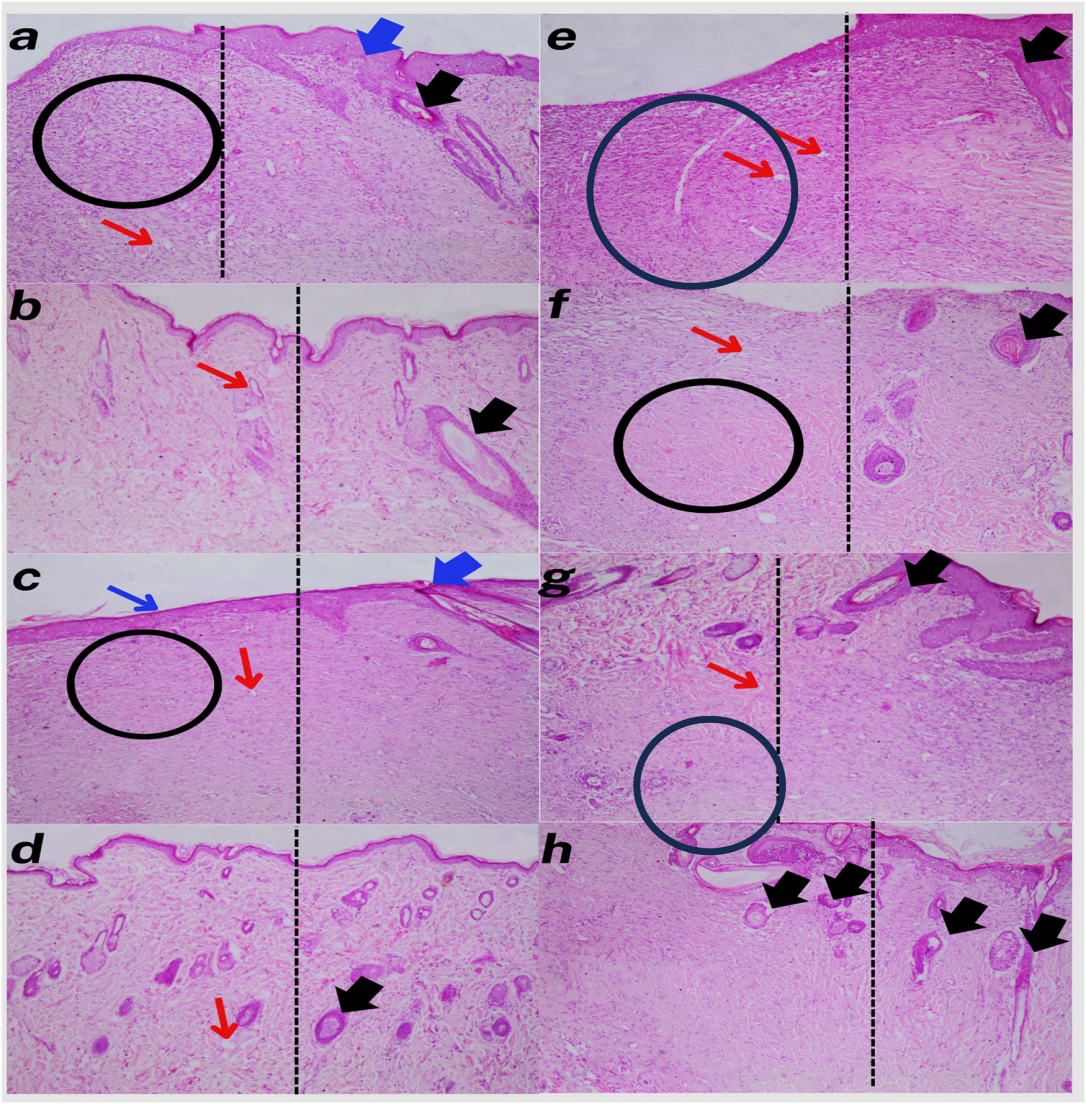
Representative photomicrographs of H&E- stained skin tissue sections at 14 days’ post-surgery. Each black dotted line in all images indicates the boundary between the wound (left) and the surrounding normal intact skin (right). Stratified squamous keratinized epithelium **(blue arrows)**. The glandular structure along with hair follicles **(black arrows)**. Newly formed blood vessels **(red arrows)**. Inflammatory cells **(circle)**. **a** control, **b** PCL, **c** HAP@PCL, **d** AgVO_3_@PCL, **e** AgVO_3_-HAP @PCL, **f** AgVO_3_/GO@PCL **g** HAP/GO@PCL, and **h** AgVO_3_-HAP/GO@PCL (10X)

**Fig. 13:**
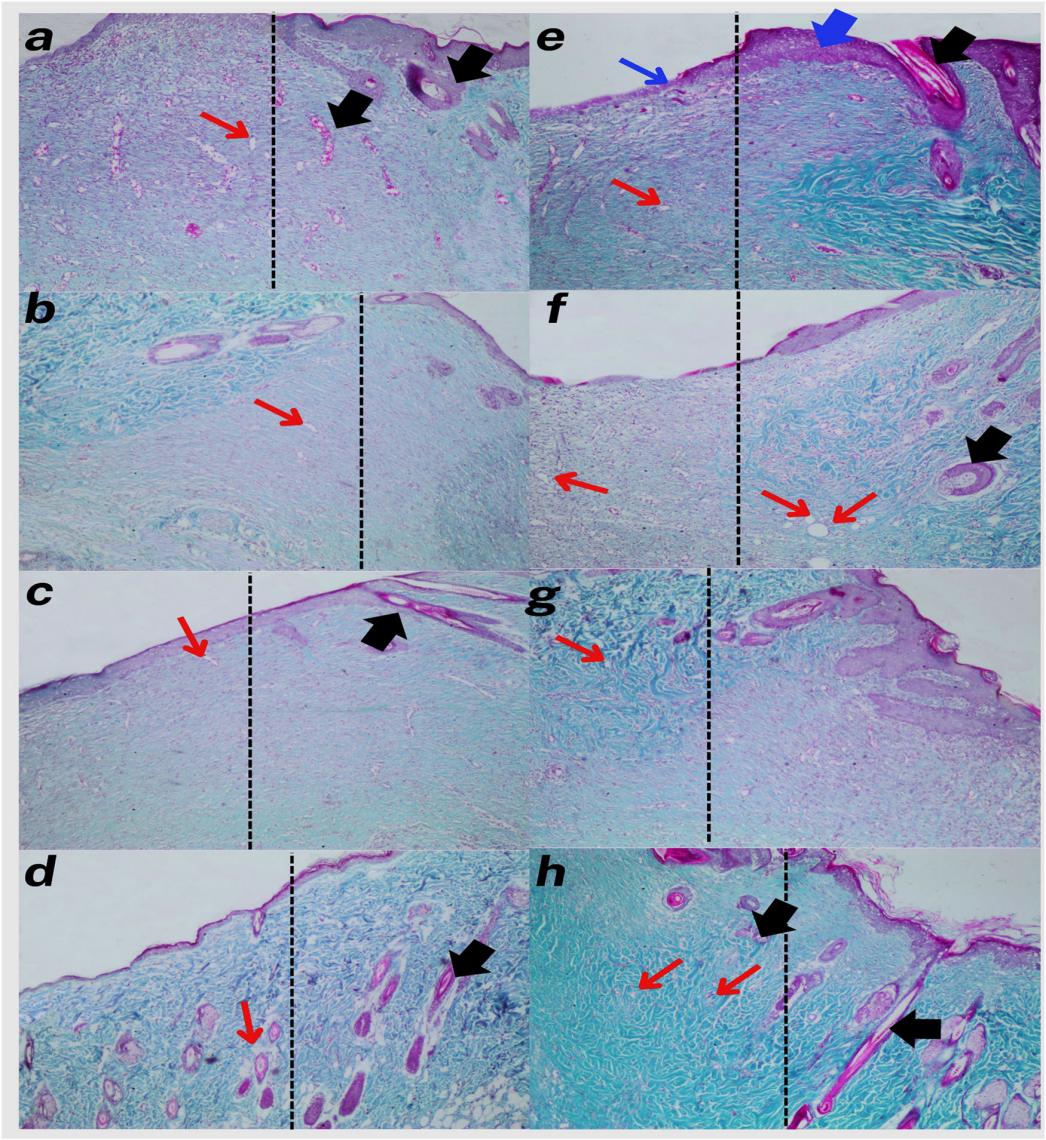
Representative photomicrographs of Masson’s trichrome-stained skin tissue sections at 14 days’ post-surgery. Each black dotted line in all images indicates the boundary between the wound (left) and the surrounding normal intact skin (right). Stratified squamous keratinized epithelium **(blue arrows)**. The glandular structure along with hair follicles **(black arrows)**. Newly formed blood vessels **(red arrows)**. Scale bars=50 µm **a** control, **b** PCL, **c** HAP@PCL, **d** AgVO_3_@PCL, **e** AgVO_3_-HAP @PCL, **f** AgVO_3_/GO@PCL **g** HAP/GO@PCL, and **h** AgVO_3_-HAP/GO@PCL (10X)

Histological analysis of skin samples from the experimental groups showed distinct differences in both cellular architecture and tissue organization, as illustrated in Figs. 12 and 13. Varying degrees of epidermal and dermal integrity, collagen deposition, vascularization, and hair follicle presence were observed across the groups.

Histological sections from the control group displayed a thin epidermis with poor cellularity (Fig. 12a) and minimal collagen deposition (Fig. 13a). The dermis appeared atrophic, with sparse vascularization and a lack of hair follicles (Fig. 12a). Additionally, the overall tissue structure indicated inadequate healing and regeneration of the tissue. The PCL group showed slight improvements in epidermal thickness and cellularity compared to the control group. However, collagen deposition remained low (Fig. 13b), and vascularization was inadequate (Fig. 12b). Histological analysis of the HAP@PCL group indicated a moderate increase in epidermal thickness and collagen density. The presence of hair follicles was also noted [79]. the overall tissue organization was still suboptimal (Fig. 12c). In contrast, the AgVO_3_@PCL group exhibited further improvements in epidermal and dermal thickness, with increased collagen deposition (Fig. 13d). Vascularization was enhanced compared to the PCL and HAP@PCL groups, but hair follicle presence remained limited (Fig. 12d). Histological sections of the AgVO_3_-HAP@PCL group revealed a thicker epidermis and dermis (Fig. 12e), with significant collagen deposition (Fig. 13e). Vascularization was improved, and hair follicles were more prominent than in the previous groups (Fig. 12e). The AgVO_3_/GO@PCL group showed histological improvement in the epidermis, increased cell density (Fig. 12f), collagen density (Fig. 13f), dermal thickness, vascularization, and prominent hair follicles.

The HAP/GO@PCL group exhibited a well-defined epidermis, increased collagen density, and enhanced vascularization. Hair follicles were present, indicating effective regeneration (Fig. 12g). Histological analysis of the AgVO_3_-HAP/GO@PCL group demonstrated the most advanced regenerative characteristics, with a thick epidermis and dermis, abundant collagen deposition, and a high number of vessels and hair follicles. The group exhibited a well-organized tissue structure. (Fig. 12h)

#### Quantitative assessment

**Fig. 14:**
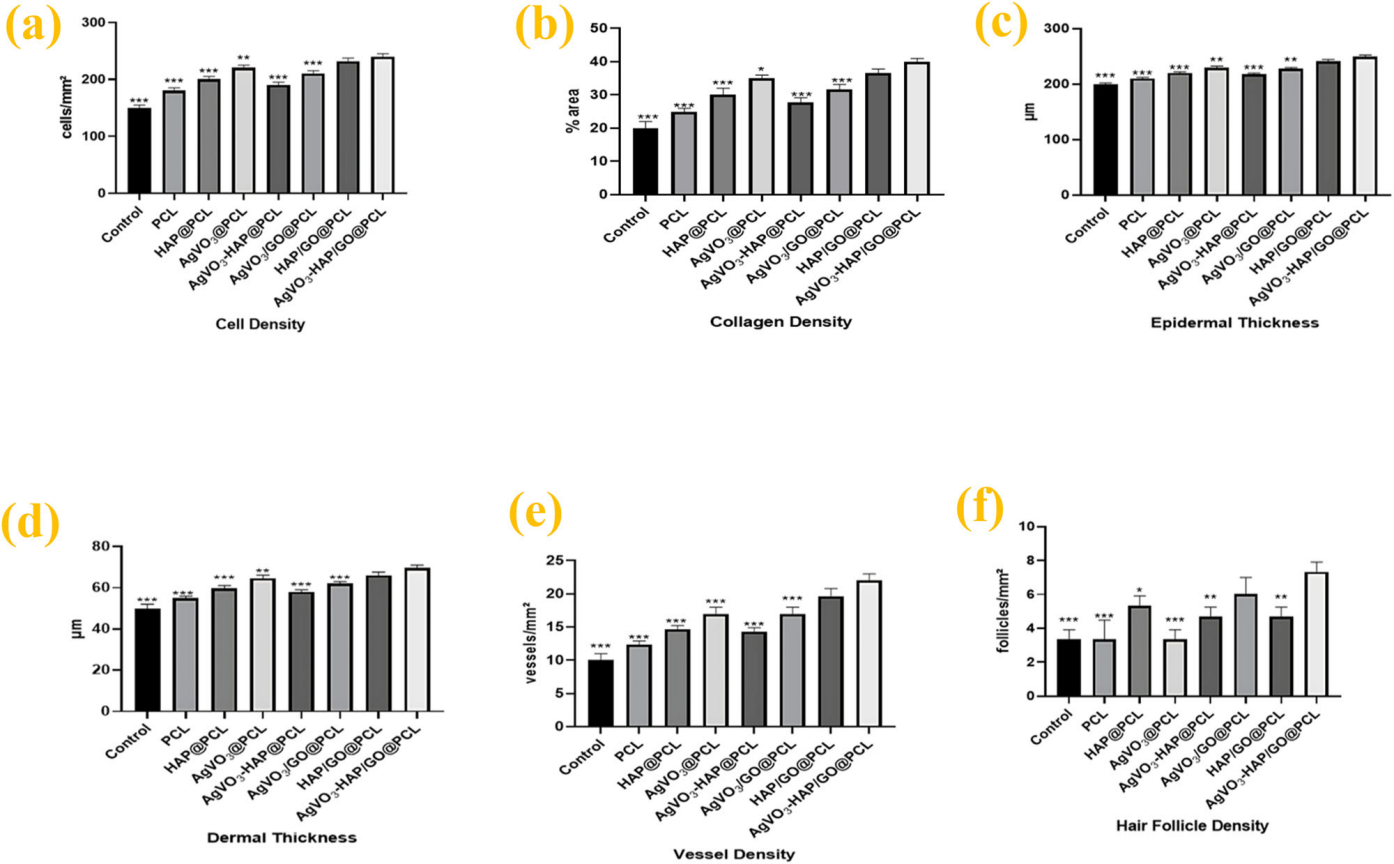
Quantitative histological analysis of wound healing parameters across different scaffold groups :**a** Cell density, **b** collagen density, **c** epidermal thickness (µm), **d** epidermal thickness, **e** vessel density, and **f** hair follicle density

Cell density, collagen density, epidermal thickness, dermal thickness, vessel density, and hair follicle density were measured in regenerated skin at day 14 after wound as showed in Fig. 14. These parameters provide insights into the regenerative capacity and healing efficacy of the skin using different scaffolds in the present experimental study. Cell density is a critical indicator of tissue regeneration and healing processes. In the present study, The AgVO_3_-HAP/GO@PCL group exhibited a significantly higher cell density than the control group (*p* < 0.001) (Fig. 14a), indicating enhanced cellular proliferation within the scaffold, which is essential for wound healing. Collagen is a fundamental component of the extracellular matrix and plays a vital role in providing structural integrity to the skin. The collagen density in the AgVO_3_-HAP/GO@PCL group was significantly elevated (*p* < 0.001) (Fig. 14b). This finding indicates that AgVO_3_-HAp/GO@PCL not only promoted fibroblast activity but also facilitated the synthesis of collagen fibers, which are crucial for restoring the mechanical properties of the skin after injury.

Epidermal thickness is a key indicator of skin regeneration. AgVO_3_-HAp/GO@PCL showed the greatest epidermal thickness, which was significantly greater than that of the control group (*p* < 0.001) (Fig. 14c). Increased epidermal thickness is often associated with improved barrier function and protection against environmental insults, further supporting effective healing in this group.

Dermal thickness is another important parameter that reflects the structural integrity of the skin. AgVO_3_-HAp/GO@PCL exhibited the highest dermal thickness (*p* < 0.001) (Fig. 14d). This increase in dermal thickness is likely due to fibroblast proliferation and collagen and other extracellular matrix component deposition, contributing to the overall strength and resilience of the healed skin.

Vascularization is crucial for delivering nutrients and oxygen to heal the tissues. Vessel density was also significantly increased in the AgVO_3_-HAp/GO@PCL group. Increased vessel density facilitates improved blood supply, which is essential for supporting metabolic demands during the healing process and promoting tissue regeneration (Fig. 14e). The presence of hair follicles is indicative of successful skin regeneration and restoration of skin appendages. Notably, the hair follicle density in the AgVO_3_-HAp/GO@PCL group was significantly higher than that in the control group (*p* < 0.01) a *p*-value < 0.001(Fig. 14f). This suggests that the treatment not only promotes healing but also supports hair follicle regeneration, which is a critical aspect of skin restoration.

The integrated histological observations and quantitative data indicate that AgVO_3_-HAp/GO@PCL group demonstrated superior skin regeneration capabilities compared to the other groups. The combination of increased cell density, collagen deposition, epidermal and dermal thickness, vascularization, and hair follicle presence highlights the effectiveness of the treatment in this group. These findings provide a comprehensive understanding of the regenerative processes involved and set the stage for further exploration of therapeutic strategies to enhance skin healing

## Conclusions

This study demonstrates the successful design and development of multifunctional electrospun scaffolds composed of PCL integrated with AgVO_3_, HAp, and GO for advanced wound healing applications. The incorporation of these bioactive nanomaterials imparted the scaffolds with superior mechanical strength, enhanced hydrophilicity, and potent antibacterial properties. Among the composites, AgVO_3_-HAp/GO@PCL exhibited a remarkable Young’s modulus of 2.31 MPa, improved ultimate tensile strength, and the highest wettability (contact angle: 65.58° ± 5.97), highlighting its suitability for dynamic wound environments. In vivo evaluations in a full-thickness rat wound model confirmed accelerated tissue regeneration, with complete wound closure achieved by day 14, supported by robust collagen deposition and re-epithelialization.

Furthermore, the scaffolds effectively inhibited both *S. aureus* and *E. coli*, underscoring their role in infection prevention. The nanofibrous architecture, resembling the extracellular matrix, provided an ideal microenvironment for cellular adhesion and nutrient diffusion. These synergistic effects position AgVO_3_-HAp/GO@PCL as a promising ceramic-polymer hybrid scaffold for next-generation wound dressings. AgVO_3_-HAp/GO@PCL can be effectively used in regenerative medicine and chronic wound management.

## Data Availability

The data underlying the findings of this study can be accessed upon reasonable request from the corresponding author. The authors are committed to maintaining transparency and promoting open science. We are willing to share the necessary data to support the validation and replication of the results presented in this paper, encouraging further research and reinforcing the reliability of our findings.
